# COPD and Depression Analysis in Regard to Obstructive Pulmonary Levels

**DOI:** 10.3390/healthcare11081175

**Published:** 2023-04-19

**Authors:** Ivana Jelić, Goran Mihajlović, Filip Mihajlović, Nataša Minić, Miloš Ratinac, Maja Pantović-Stefanović

**Affiliations:** 1 Faculty of Medical Sciences, University of Kragujevac, 34000 Kragujevac, Serbia; 2 Clinic of Psychiatry, University Clinical Center Kragujevac, 34000 Kragujevac, Serbia; 3 Clinic of Pulmonology, University Clinical Center Kragujevac, 34000 Kragujevac, Serbia; 4 Faculty of Medicine, Department of Psychiatry, University of Belgrade, 11000 Belgrade, Serbia; 5 Clinic of Psychiatry, University Clinical Center of Serbia, 11000 Belgrade, Serbia

**Keywords:** chronic obstructive pulmonary disease, depression, Hamilton scale, Serbia

## Abstract

Depression symptoms take place recurrently in patients suffering from COPD. This study aims to assess the effects of antidepressant therapy in patients with COPD and a depressive disorder in relation to COPD levels. The study population consisted of N = 87 patients diagnosed with COPD, according to the GOLD criteria, and a depressive disorder. All of the patients were subjected to clinical and psychiatric exploration according to psychiatric assessment instruments, which was followed by SSRI therapy for the duration of 8 weeks. The main methods used were descriptive statistics and analysis of variance. The results showed a different distribution of depressive symptoms at a different stage of COPD by FEV1 (χ^2^ = 30.47, df = 6, *p* < 0.01) and by mMRC (χ^2^ = 34.6, df = 6, *p* < 0.01). After the application of SSRIs, there was a significant improvement in HDRS scores in all stages of COPD by FEV1 (χ^2^ = 251.62, df = 9, *p* < 0.01) and by mMRC (χ^2^ = 919.17, df = 9, *p* < 0.01). This study contributes to the improvement in the quality of life of patients by the targeted application of SSRI therapy and, therefore, more precise and better overall treatment results.

## 1. Introduction

Chronic obstructive pulmonary disease (COPD) is a common respiratory disease [1] and a severe health problem, especially when the primary disease is complicated by depression symptoms whose treatment requires a highly complex therapeutic approach [2,3]. It has been shown in several studies that depression symptoms take place quite often in patients suffering from COPD, and prevalence ranges from 18% to 84% [4,5,6,7]. The variability of these symptoms in studies stems from the differing stage of the disease, as well as various diagnostic COPD criteria. There exist numerous factors that contribute to the development of COPD [8], such as the consumption of nicotine [9,10,11,12,13,14,15].

Previous studies have indicated that COPD is often accompanied by symptoms of depression. Insofar as their symptoms are intertwined, it is difficult to distinguish between depression and COPD [16]. A large number of mental and physical symptoms are related to disorders such as increased fatigue, sleep and appetite disorders, difficulty with concentration, and difficulty with movement [17,18]. People with depression smoke more often and more intensely; therefore, depression negatively affects smoking cessation. Depression is a significant risk factor for frequent COPD exacerbations, shortening the time between two exacerbations [19]. Additionally, the presence of depression makes it difficult to accept specific types of treatment for advanced forms of COPD, such as non-invasive mechanical ventilation [20]. The treatment of depression in patients with COPD requires a multidisciplinary approach [21]. However, there is a lack of research on the effects of treating depression in patients with COPD, e.g., [7,22,23], particularly in Balkan countries such as Serbia, e.g., [24,25].

Thus, this study was particularly designed to assess the effects of antidepressant therapy on patients with COPD and a depressive disorder in relation to COPD levels.

## 2. Materials and Methods

### 2.1. Participants

This research is a prospective cohort study involving primary care patients treated with COPD with the comorbidity of depression. The study population consists of N = 87 patients diagnosed with COPD according to the Global Initiative for COPD (GOLD) criteria [26]. Patients treated with COPD were categorized into groups concerning the severity of the disease. The severity of the disease was based on the post-bronchodilator forced expiratory volume in 1 s (FEV1) [27] and was grouped in relation to the modified Medical Research Council dyspnea scale (mMRC) for dyspnea to degrees (levels) 0–3 [28]. The first group of patients consisted of patients with mild COPD (stage 1, FEV1 ≥ 80% of predicted value), the second group consisted of patients with moderate COPD (stage 2, 50% ≤ FEV1 < 80% of predicted value), the third group consisted of patients with severe COPD (stage 3, 30% ≤ FEV1 < 50% of predicted value), and the fourth group consisted of very severe COPD patients (stage 4, FEV1 < 30% of predicted value). The clinical diagnosis of depression in patients suffering from COPD was set by a psychiatrist in primary care institutions in accordance with the ICD-10 criteria for Major Depression (F32.2). Selective serotonin reuptake inhibitor (SSRI) therapy was prescribed by a psychiatrist in a primary care setting following good clinical practice guides. The selection of particular SSRIs was made according to the guidelines of good clinical practice, and the selection was left to the treating psychiatrist to decide upon in regard to the patient’s condition.

Ethics Statements. Epidemiological monitoring of the effects of antidepressant therapy on the psychophysical and social functioning of patients with COPD and symptoms of depression as a comorbidity was conducted in primary care patients of the Kragujevac Health Center (Serbia) in the period from October 2016 to December 2019, following the decision of the Ethics Committee of the Kragujevac Health Center (01-542/2). All patients provided written informed consent before their inclusion in the study.

### 2.2. Methods

All the subjects included in the study were diagnosed with COPD and depression as a comorbidity and were subsequently subjected to treatment with SSRI therapy. Along with the demographic questionnaire used to collect general socio-demographic data (gender and age), the following psychiatric assessment instruments, which have already been used in previous studies in Serbia, were also used:

1. Patient Health Questionnaire-9 (PHQ-9);
2. Quality of Life Enjoyment and Satisfaction Questionnaire–Short Form (Q-LES-Q-SF);
3. Hamilton Depression Rating Scale-17 (HDRS).

During the survey, all participants filled out the questionnaire to assess depression symptoms. The questionnaire was created by licensed psychiatrists for the purpose of screening depression symptoms in primary health care. The instrument was physician-administered and used to initially screen the patients for depression. The Q-LES-Q-SF was used to assess quality of life enjoyment and satisfaction on the following scale: very bad, bad, average, and good. This instrument was used to collect socio-demographic data, such as housing status, economic status, and work. The following categories of data were collected: psychophysical and social functioning; physical functioning in relation to mMRC before and after the use of SSRIs; and psychosocial and physical functioning in relation to FEV1 before and after the use of SSRIs. The HDRS [29] clinician-rated psychometric instrument with 17 items was used to assess the severity of depression symptoms. To grade the severity of depression, the patients were divided into groups according to the HDRS score as follows: values from 0 to 13: without depression; values from 14 to 19: mild depression; values from 20 to 28: moderate depression; and values from 29 to 63: expressed depression.

All patients underwent a complete psychiatric workup, which consisted of complete psychiatric history, corroborated hetero-anamnestic data, major problems, present illness, and personal and family history. They were subjected to clinical and psychiatric exploration according to psychiatric assessment instruments. This was followed by SSRI antidepressant therapy. SSRIs are a class of antidepressants that use the inhibition of the reuptake of serotonin to neuron as a mechanism of action to increase serotonin levels in the synaptic cleft. They usually cause fewer side effects compared to other classes of antidepressants, are relatively safe, and are thus administered frequently [29]. In the study, they were applied in accordance with the current guidelines for the duration of 8 weeks, followed by SSRI antidepressant therapy. After 8 weeks, the listed procedures from the first visit were repeated (psychological status and scale assessment). The exclusion somatic criteria were type 1 diabetes mellitus, malignant diseases, ventricular arrhythmias, ischemic heart disease, and chronic renal or liver failure. The exclusion mental disorders were any other mental disorder. During the study, no specific programs (i.e., pulmonary rehabilitation, smoking cessation, dietician consultation, drug modification, or psychological support) were applied.

In total, 604 patients with COPD were identified during the study period. All the patients were invited to participate in the study. Out of the total number of identified patients, 522 agreed to participate in the study, and they provided written informed consent. Only the patients with COPD and comorbid depression, 126 in total, were selected to further participate in the study. An additional 19 patients were excluded due to the presence of another mental disorder apart from depression. Using exclusion somatic criteria, the patients were further excluded from the study (type 1 diabetes mellitus: 8; malignant diseases: 0; ventricular arrhythmias: 2; ischemic heart disease: 4; and chronic renal or liver failure: 2). Finally, 3 patients were excluded after the initiation of SSRIs due to the adverse effect of antidepressants, and 1 patient was lost to follow-up, which derived a final sample of 87 subjects who completed the study.

### 2.3. Statistical Analysis

The data used were defined by descriptive statistics and analyzed using adequate statistical methods. Depending on the variable type, the results were expressed as numbers and percentages (categorical variables) and mean ± SD (continuous variable). The mean age of the patients in a different stage of disease according to FEV1 and mMRC was tested using ANOVA (analysis of variance). Differences in the frequency of the level of depressive disorder in relation to the severity of the underlying disease and the distribution of different HDRS levels by the stage of COPD before and after the use of SSRI therapy were tested. The analysis relied on χ^2^. The Statistical Package for Social Sciences (SSPS) (Version 19.0) was used for the analysis and the statistical procedure.

## 3. Results

### 3.1. Sample Characteristics

A total of 87 patients with COPD and depression were included in the study with a mean age of 48.84 ± 7.43 (range 34–62). The majority of the study population was made up of females (N = 57, 65.5%) rather than males (N = 30, 34.5%). Additionally, 36.8% of the patients self-reported as poor, and 36.8% reported an average economic status (i.e., not rich, but not poor either). The majority of patients (93.1%) assessed their housing situation to be normal (possessing their own house).

### 3.2. COPD Stages in Patients

Table 1 shows the general characteristics of the patients at different stages of COPD according to FEV1 and mMRC. The analysis using the FEV1 stage of the disease showed the different number of patients who were in a different FEV1 stage (χ^2^ = 89.14, df = 3, *p <* 0.01). Two thirds of patients were at stage II of the disease. The average age of the patients in the sample was 48.8 ± 7.4. There was a significant difference in the age of patients according to the FEV1 stage (F = 3.95, df = 3, *p* < 0.05); the oldest patients were at stage II of the disease. FEV1 stages by sex were similar for both genders (χ^2^ = 0.67, df = 3, *p* > 0.05).

The Modified Medical Research Council Dyspnea Scale showed that every third patient was at level 1, and every fourth patient was at level 2 of the disease (χ^2^ = 11.35, df = 3, *p* < 0.05). The youngest patients were at level 1, and the oldest were at the level 3 disease stage (F = 6.29, df = 3, *p* < 0.05). The patients with mMRC level 1 were the youngest, and patients with mMRC level 3 were the oldest. The mMRC level was similar for both genders (χ^2^ = 2.38, df = 3, *p* > 0.05) (Table 1).

### 3.3. Depression in COPD Patients before and after Therapy

Table 2 shows the analysis of the frequency of the severity of a depressive disorder (graded 0–3 according to the group scores on HDRS) in regard to the FEV1 stage and mMRC level before and after SSRI therapy.

**Figure 1:**
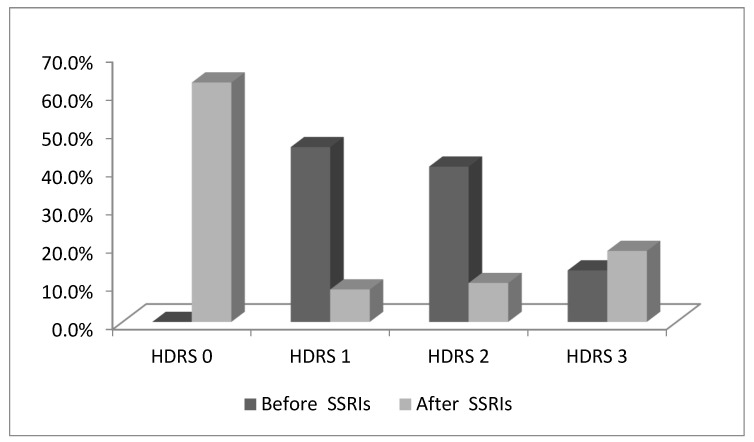
Effect of SSRIs on stage II COPD.

Given the heterogeneity of the study population and the small sample size, we focused on stage II COPD to analyze the effect of SSRIs. A clear difference in regard to a change in depression symptom severity, grouped according to HDRS score, was observed after SSRI treatment (χ^2^ = 29.98, df = 6, *p* < 0.05) in this group of patients. Before treatment, the largest number of patients belonged to groups 1 or 2 according to HDRS score, while there were no patients in group 0. After SSRI therapy, more than half of the patients were in group 0 according to HDRS score. More patients at stage II of COPD progressed from HDRS 2 to HDRS 3 after the administration of SSRIs (Figure 1).

At the beginning of the study (before SSRI therapy), there was a statistically significant difference in the level of HDRS compared to the stage of COPD according to FEV1 (χ^2^ = 30.47, df = 6, *p* < 0.01). In view of that, every second patient with FEV1 stage II was at level 1 of HDRS before SSRI, as well as every fourth patient, who had the easiest form of obstruction. All the patients who were at level 2 of HDRS before SSRI were at stage II of COPD, and the most severe form of depressive disorder was seen in patients who were at stage II and III. After 8 weeks of therapy, there was a statistically significant improvement (χ^2^ = 251.62, df = 9, *p* < 0.01). After SSRI therapy, most patients, regardless of the severity of the underlying disease, were at level 0 of HDRS. Level 1 of HDRS after SSRI was found in patients at stages II and III, level 2 at stages I and II, while the most severe form (level 3) of depressive comorbidity was found at stage II of FEV1.

According to HDRS before SSRI, level 0 was not present in any patient, while level 1 was most commonly diagnosed in mMRC-1 (61.7%) (Table 2). Every second patient with mMRC-0 and every third with mMRC-2 were at level 2. The most severe form of depression is most often diagnosed in individuals at the most severe stage of the disease. After the application of antidepressant therapy, there was a statistically significant improvement (χ^2^ = 919.17, df = 9, *p* < 0.01). The largest number of patients (over 70%), at all levels of mMRC, had the lowest degree of depression, while 12.6% of patients with all stages of dyspnea were diagnosed with level 3 HDRS after SSRI (Table 2).

Table A1 (Appendix A) shows the results of the psychophysical and social functioning characteristics according to mMRC before and after SSRI therapy based on the assessment of the patients themselves. The number of patients who rated social behavior as bad significantly decreased after SSRI therapy (χ^2^ = 1200.2, *p* < 0.01). Thus, the percentage of patients at stage I or II of COPD, and who rated their social behavior as bad, decreased from 31%, and 24.1%, respectively, to 1.1% after SSRI therapy. Although the patients were undergoing therapy for 8 weeks, no significant difference (decrease) in the number of those who rated their family behavior as bad was observed (*p* > 0.05). However, according to mMRC, after the application of SSRIs, there was a significant increase in how the patients viewed their wellbeing, regardless of the severity of their underlying disease (χ^2^ = 1551.7, *p* < 0.01).

Table A2 (Appendix A) shows the physical functioning characteristics. Overall, SSRI therapy had a positive effect on free activity. Although at the beginning of the study a large number of patients (from all stages of underlying disease) assessed free activity as bad, after SSRI therapy, only one patient at stage 0 and one at stage II of COPD rated their free activity as bad (χ^2^ = 586.1, *p* < 0.01). Treatment of depressive disorder had a significant effect on the increase in functioning in daily life. Although at the beginning the largest number of patients rated functioning in their daily life as bad, 8 weeks after SSRI therapy, only a few patients retained their previous attitude (χ^2^ = 812.2, *p* < 0.01).

Table A3 (Appendix A) shows the characteristics of psychosocial functioning according to the stage of disease (FEV1) before and after the use of SSRIs. Before SSRIs, most patients at stage I of disease rated their social behavior as bad. After therapy, the majority of patients felt that they had improved (χ^2^ = 666.8, *p* < 0.01). Eight weeks after therapy, no significant change in family behavior was observed (*p* > 0.05). Sense of wellbeing, which before taking SSRIs was rated by the largest number of patients, especially at stage I, as bad, also improved after treatment of a depressive disorder as only a few patients at stage I and stage II retained their previous opinion (χ^2^ = 588.8, *p* < 0.01).

Table A4 (Appendix A) shows the results of physical functioning according to FEV1 before and after the use of SSRIs. SSRIs significantly affected the physical functioning of patients in all stages of COPD, i.e., free activities (χ^2^ = 84.5, *p* < 0.01) and functioning in everyday life (χ^2^ = 606.3, *p* < 0.01). Before starting treatment for a depressive disorder, every second patient at stage I of the disease rated their functioning in everyday life as bad. After SSRIs, none of these patients thought that their free activity was bad. Functioning in daily life after therapy was rated by only a few patients as bad.

## 4. Discussion

In this study, a total of 87 subjects with COPD were diagnosed with a depressive disorder (65.5% were women), which is partially in accordance with the epidemiological data of COPD subjects in Kuehne’s study [30]. Comorbid depression in patients was treated with antidepressants, and the outcome of the therapy indicated a statistically significant difference in relation to the severity of COPD. Patients with symptoms of mild depression were treated with antidepressants, and this accounted for a total of 12% of patients. In the group of patients with moderate depression, the depression of the treated SSRIs with antidepressants totaled 10.2% of patients. In the group of patients with marked depression, the depression of the treated SSRIs with antidepressants accounted for a total of 7.4% of patients. There is a statistically significant difference in the distribution of depression in patients with COPD who were treated by SSRIs with antidepressants compared to the severity of COPD according to the GOLD criteria. Namely, after antidepressant treatment, a redistribution to lesser symptom categories was observed.

Carvalho and colleagues [31] found that the percentage of women who suffered from COPD with depression comorbidity was 45% of the total number of their subjects. In a 2018 study [32], the average age of patients with COPD diagnosed with depression was 57.8, which is in line with our research results. Another 2018 study [4] noted that the average age of patients with COPD in whom a depressive disorder was diagnosed was 44. In another study [19], whose authors also looked at patients with COPD with diagnosed depressive disorder, the average age of the patients with COPD with comorbid depression was 57 years. Our study included patients below 50 years, correlating with [4] and [19]. Although the patients were almost a decade younger compared to the average age of patients with COPD diagnosed with depression, it is notable that the exclusion criteria were rigorous. The exclusion of patients with additional comorbidities from this and similar studies is of particular value as depression has been repeatedly linked to a variety of somatic disorders influencing one’s general medical condition [33], which improved the precision of the results derived from our study.

By analyzing the correlation in this research, it was found that the mean value of the degree of depression was the same as the age of patients in relation to the degree of dyspnea in patients with COPD in whom depressive diagnosis was poor (F = 6.29, df = 3, *p* < 0.05), which is in accordance with Bratek et al., who claimed in their study that the level of depression was correlated with the level of COPD and the age of patients was correlated with the level of dyspnea [21].

In this study, there were N = 87 patients with COPD with depression comorbidity, which, according to the GOLD criteria, were grouped into stages. Likewise, most patients were at the GOLD-2 (67.8%), GOLD-3 (17.2%), GOLD-1 (12.6%), and GOLD-4 (2.3%) stages of the disease. In another study [32], N = 202 patients with COPD with depression comorbidity were included. According to the GOLD criteria, 13.4% of patients were at the GOLD-1 stage of the disease, 43.07% were at the GOLD-2 stage, 29.21% were at the GOLD-3 stage, and 14.36% were at the GOLD-4 stage, which is very similar to our results, differing only by a significantly smaller percentage of patients at the GOLD-1 stage. This is consistent with epidemiological studies that show that the largest number of patients with COPD are at stage 1 and 2 of the disease [34].

The most common respiratory symptom in the population was dyspnea. In this study, patients were grouped in relation to mMRC. Most patients had mMRC-1 of 38%, mMRC-2 of 26.4%, mMRC-0 of 23%, and mMRC-3 of 12.6%. When analyzing the correlation of variables with the HDRS scale, the average HDRS depression rate in the group of patients with COPD treated with SSRIs as antidepressants was mMRC-0 of 20, with mMRC-1 of 52, mMRC-2 of 22.5, and mMRC-3 of 5.5. It is notable that not all patients had a positive response to treatment, which could be attributed to various reasons. The follow-up period used in the study is in accordance with the current guidelines and an adequate time for the patients to show treatment response. However, the remission period could be entered later and although they showed symptom improvement, not all patients were able to achieve remission during the follow-up period. Additionally, in our study, the most severe form of depression was most often diagnosed in those individuals at the most severe stage of the disease, before treatment, which could additionally complicate the treatment response as it has been noted that patients in more progressed forms of COPD tend to recover slower [35]. Moreover, previous studies have also found that only a minority of patients respond to the antidepressant of their first choice [36].

This study shows that physical condition, work ability, and social interaction are three major problems for patients. In the treatment of these patients, the medications used to normalize the symptoms of depression had a statistically significant effect on their quality of life. Epidemiological studies addressing this issue show that the introduction of SSRI therapy is positively correlated with the quality of life of subjects, as confirmed by this study. The mechanism through which antidepressants regulate patients’ mood, in SSRI treatment, is the selective inhibition of serotonin reuptake, which results in the improvement in the symptoms of depression [37,38].

The study has some limitations and strengths that should be considered when interpreting the results. First, the study sample is modest, and the study applied one group of antidepressants, but not the same antidepressant, which limits the generalization of the results. However, previous studies exploring the effects of antidepressant treatment on COPD outcome in depressed COPD patients had relatively small sample sizes, suffered from high dropout rates, and focused only on one group of antidepressants or failed to conduct a follow-up with patients for a significant period of time [39]. We additionally focused on patients with stage II COPD to meet the challenge of clinical heterogeneity of the study population. To increase comparability, this study used psychometric instruments that have been used in similar previous studies and that are considered valuable and reliable “gold standards” in psychopathology assessment. It is notable that some instruments were used to improve the screening process and not to assess the severity of the disorder, which could have been of additional value to the study.

Clinical studies that demonstrate the beneficial effects of antidepressant medication are oftentimes designed by excluding significant somatic disorders. The advantage of this study is that by specifically assessing the effects of antidepressants in relation to the severity of pulmonary symptoms in COPD, it provides new and important data on this comorbidity. As such, this study fills a gap in the literature and addresses a key question in contemporary medicine, namely the comorbidity of psychiatric and somatic disorder.

## 5. Conclusions

This study shows that in a clinical population of patients with COPD, depression can significantly affect the improvement of COPD. In the population of middle-aged patients in all stages of COPD, SSRIs significantly improved patients’ physical functioning (i.e., level 0 of HDRS after SSRI in over 70% of patients), as well as their free activities and functioning in everyday life.

The symptoms of depression in COPD patients are at a very high level and directly affect the deterioration of the patient’s quality of life and, consequently, the worsening of the somatic disease.

Given the World Health Organization’s prediction that depression will become the leading cause of morbidity in the world by 2030, and that it is already one of the health conditions most impacting decline in quality of life [40], adequate prevention and promotion of the role of consultative psychiatry is becoming imperative.

Patients suffering from chronic obstructive pulmonary disease fall into the ideal category for preventative work, primarily because they are a high-risk population and because they are in constant contact with healthcare professionals, which facilitates conducting screenings.

The results obtained through this research represent a significant step towards improving patients’ quality of life with the use of SSRIs, and, thus, the improvement of the overall outcome of treatment for COPD patients.

Although the study was conducted with a large population to relevantly examine the association between depression symptoms and the impact of antidepressant therapy in patients with COPD and a depressive disorder as co-morbidities in relation to COPD levels, it is necessary to conduct a new study in the foreseeable future to determine the impact of other types of antidepressants on COPD subjects, in addition to monitoring the effects of SSRIs and comparing the obtained results with a control group.

## Figures and Tables

**Table 1:** The general characteristics of patients at different stages of COPD.

FEV1/mMRC	n (%)	*p*	Age	*p*	Sex (n, %)		*p*
			$\left(\bar{x}\;\pm \;\mathrm{sd}\right)$		Female	Male	
**FEV1**							
Stage I	11 (12.6)	<0.01	42.2 ± 6.02	<0.05	8 (9.2)	3 (3.4)	>0.05
Stage II	59 (67.8)		49.9 ± 6.6		39 (44.8)	20 (23)	
Stage III	15 (17.2)		44.7 ± 9.6		9 (10.3)	6 (6.9)	
Stage IV	2 (2.3)		42 ± 1.4		1 (1.1)	1 (1.1)	
**mMRC**							
Level 0	20 (23)	<0.05	51.2 ± 5.9	<0.05	13 (14.9)	7 (8)	>0.05
Level 1	33 (37.9)		44.9 ± 6.9		23 (26.4)	10 (11.5)	
Level 2	23 (26.4)		50.7 ± 6.9		16 (18.4)	7 (8)	
Level 3	11 (12.6)		52.7 ± 7.7		5 (5.7)	6 (6.9)	

Note: $\bar{x}$ is the mean and SD is the standard deviation of the mean.

**Table 2:** Severity of depressive disorder (graded according to group scores on HDRS) levels before and after SSRI therapy according to FEV1 and mMRC.

Variables		HDRS (%)							*p*
		Before SSRIs			After SSRIs				
	0	1	2	3	0	1	2	3	
**FEV1**									
Stage I	0	12.6	/	/	9.2	/	3.4	/	<0.01
Stage II	0	31	27.6	9.2	42.5	5.7	6.9	12.6	
Stage III	0	8	/	9.2	16.1	1.1	/	/	
Stage IV	0	2.3	/	/	2.3	/	/	/	
**mMRC**									
Level 0	0	5.7	12.6	4.6	16.1	2.3	2.3	2.3	<0.01
Level 1	0	33.3	3.4	1.1	29.9	1.1	3.4	3.4	
Level 2	0	11.5	9.2	5.7	12.6	3.4	3.4	6.9	
Level 3	0	3.4	2.3	6.9	11.5	/	1.1	/	

## Data Availability

The datasets used and analyzed during this study are available from the corresponding author upon reasonable request. The data were not publicly available because of ethical considerations.

## Appendix A

The appendix contains Table A1, Table A2, Table A3 and Table A4 cited in the main text. The results presented in the tables are discussed in the main text, while the table presentation is below, in order to not disrupt the flow of the main text.

**Table A1:** Characteristics of psychophysical and social functioning (bad) according to mMRC before and after the use of SSRIs.

Variable	Before SSRIs		After SSRIs	*p*
	(n, %)		(n, %)	
**Social behavior**				
Level 0	16 (18.4)		5 (5.7)	<0.01
Level 1	27 (31)			
Level 2	21 (24.1)		1 (1.1)	
Level 3	11 (12.6)		1 (1.1)	
**Family behavior**				
Level 0	20 (23)		19 (21.8)	>0.05
Level 1	27 (31)		21 (24.1)	
Level 2	21 (24.1)		15 (17.2)	
Level 3	11 (12.6)		9 (10.3)	
**A sense of wellbeing**				
Level 0	19 (21.8)	3 (3.4)		
Level 1	32 (36.8)	1 (1.1)		<0.01
Level 2	23 (26.4)	1 (1.1)		
Level 3	11 (12.6)	3 (3.4)		

Note: % of total number patients (n = 87).

**Table A2:** Characteristics of physical functioning (bad) according to mMRC before and after the use of SSRIs.

Characteristics	Before SSRIs	After SSRIs	*p*
	(n, %)	(n, %)	
**Free activity**			
Level 0	20 (23)	1 (1.1)	<0.01
Level 1	25 (28.7)	-	
Level 2	16 (18.4)	1 (1.1)	
Level 3	10 (11.5)	-	
**Functioning in daily life**			
Level 0	19 (21.8)	1 (1.1)	<0.01
Level 1	23 (26.4)	2 (2.3)	
Level 2	17 (19.5)	1 (1.1)	
Level 3	9 (10.3)	3 (3.4)	

Note: % of total number patients (n = 87).

**Table A3:** Characteristics of psychosocial functioning (bad) according to the stage of disease (FEV1) before and after the use of SSRIs.

Variable	Before SSRIs	After SSRIs	*p*
	(n, %)	(n, %)	
**Social behavior**			
Stage I	7 (8)	1 (1.1)	<0.01
Stage II	53 (60.9)	5 (5.7)	
Stage III	13 (14.9)	1 (1.1)	
Stage IV	2 (2.3)	1 (1.1)	
**Family behavior**			
Stage I	9 (10.3)	8 (9.2)	>0.05
Stage II	54 (62.1)	42 (48.3)	
Stage III	14 (16.1)	13 (14.9)	
Stage IV	2 (2.3)	1 (1.1)	
**A sense of wellbeing**			
Stage I	11 (12.6)	-	<0.01
Stage II	57 (65.5)	5 (5.7)	
Stage III	15 (17.2)	3 (3.4)	
Stage IV	2 (2.3)	-	

Note: % of total number patients (n = 87).

**Table A4:** Characteristics of physical functioning (bad) according to FEV1 before and after the use of SSRIs.

Characteristics	Before SSRIs	After SSRIs	*p*
	(n, %)	(n, %)	
**Free activity**			
Stage I	7 (8)	-	<0.01
Stage II	47 (54)	-	
Stage III	15 (17.2)	2 (2.3)	
Stage IV	2 (2.3)	-	
**Functioning in daily life**			
Stage I	10 (11.5)	1 (1.1)	<0.01
Stage II	43 (49.4)	4 (4.6)	
Stage III	13 (14.9)	1 (1.1)	
Stage IV	2 (2.3)	1 (1.1)	

Note: % of total number patients (n = 87).
